# Draft genome sequence of *Rhizobium leguminosarum* I10 isolated from root nodules of *Tephrosia purpurea*

**DOI:** 10.1128/mra.00217-26

**Published:** 2026-06-03

**Authors:** Pragadeesh Ayyamuthu Rajarathinam Uma, Marimuthu Subramanian, Senthil Kumar Murugaiyan, Dinesh Antony Vedhamuthu, Alisha Farheen, Pavan Jadav, Nanthitha Ashok, Siva Muthamizhan, Vidya de Gannes

**Affiliations:** 1 Department of Agricultural Microbiology, Tamil Nadu Agricultural University, Coimbatore, India; 2 Centre for Agricultural Nanotechnology, Tamil Nadu Agricultural University, Coimbatore, India; 3 Department of Plant Pathology, Tamil Nadu Agricultural University, Coimbatore, India; 4 Department of Food Production, The University of West Indies, Saint Augustine Campus, Saint Augustine, Trinidad and Tobago; University of Strathclyde, Glasgow, United Kingdom

**Keywords:** *Rhizobium leguminosarum*, *Tephrosia purpurea*, genome, sequencing

## Abstract

We report the draft genome of *Rhizobium leguminosarum* strain I10 (7,598,161 bp scaffolded genome length, 60.5% GC, 7,061 protein-coding genes) isolated from the root nodules of *Tephrosia purpurea*.

## ANNOUNCEMENT

*Rhizobium leguminosarum*, a gram-negative bacterium, is renowned for its symbiotic association with legumes and nitrogen fixation within root nodules. *Rhizobium leguminosarum* strain I10 was isolated from *Tephrosia purpurea* root nodules collected on 17 November 2022 at Dr. M.S. Swaminathan Agriculture College and Research Institute, Eachangkottai, Thanjavur (10.6621° N, 79.1594° E). Nodules were surface sterilized with 70% ethanol for 5–10 seconds, followed by 2% sodium hypochlorite for 1 minute, and then rinsed three times with sterile water (1, 2). Sterilized nodules (1 g) were crushed with a sterile glass rod in a test tube filled with a large drop of sterile water. The suspensions were serially diluted up to 10⁻⁶ dilutions and pour-plated onto Congo Red Yeast Extract Mannitol agar (3) and incubated aerobically at 28°C for 3–5 days. Pure cultures were obtained by repeated streaking on YEMA under the same temperature and aerobic conditions (4). To elucidate the genetic makeup of the isolate I10, whole-genome sequencing was subsequently performed.

Genomic DNA was extracted from a single purified colony grown in TY broth (tryptone, 5 g/L; yeast extract, 2.5 g/L) at 30°C for 18–24 h under shaking conditions, using a QIAGEN Genomic-tip 20/G Kit, following the manufacturer’s guidelines (5, 6). DNA quantity and integrity were assessed using a Qubit 4 Fluorometer and agarose gel electrophoresis (7). The genomic DNA was sent to ONEOMICS Private Limited (Tiruchirappalli, India) for whole-genome sequencing. Illumina libraries were prepared using the TruSeq DNA PCR-Free Kit (Illumina, Inc., USA); the genomic DNA is fragmented, end repaired, A-tailed, size selected, and ligated with Illumina sequencing barcoded adapters. Library concentration was quantified using a QuantStudio real-time PCR system (Applied Biosystems, USA), and fragment size distribution was verified on an Agilent 2100 Bioanalyzer (Agilent Technologies, Inc., USA). Sequencing was performed on the Illumina NovaSeq 6000 platform (2 × 150 bp) (Illumina, Inc.), generating 35,036,626 raw reads totaling 5,255,493,900 bp.

Raw reads were quality-checked with FastQC v0.10.1 (8) and trimmed using TrimGalore (v0.6.10) (9). Genome assembly was generated using SOAPdenovo (v.2.04) (10) and polished with Pilon (v1.19) (11). The draft genome assembly spans 7,597,499 bp with 60.5% GC content (Table 1). The draft assembly was scaffolded with RagTag (v2.1.0) using the reference genome CP071626.1, resulting in a final scaffolded genome length of 7,598,161 bp, and the genome quality was evaluated using QUAST (v5.3.0) (12). Genome completeness was 99.58%, assessed using the BUSCO v5 bacteria_odb10 lineage data set (13, 14). Gene prediction and functional annotation were performed with PGAP (v6.10) (15, 16). Strain I10 was classified as *Rhizobium leguminosarum* using GTDB-Tk (v1.7.0 reference set R202) (17, 18). The closest GTDB representative genome was *R. leguminosarum* USDA 2370 (GCF_002008365.1), with an average nucleotide identity of 98.09%. Default parameters were used for all tools unless otherwise specified.

**TABLE 1:** Detailed information of the draft genome

Feature	Value
Genome size (ungapped length) (bp)	7,597,499 bp
Number of chromosomes	1
Number of contigs	7
Contig N50 (bp)	2,311,306 bp
Contig L50	2
GC content (%)	60.5
Genome coverage	625×
BUSCO completeness (%)	99.58
SRA	SRS24631670
GeneBank assembly	CP196748

## Data Availability

The whole-genome sequence project for *Rhizobium leguminosarum* I10 has been deposited at GenBank under accession number CP196748. The raw reads are available under BioProject accession number PRJNA1247883, and the BioSample accession number is SAMN47832398. The sequence data obtained in this work have been deposited in the NCBI Sequence Read Archive under accession number SRS24631670. Additionally, the assembled genome sequence is available in GenBank under accession number GCA_051904995.1.
